# Adherence to highly active antiretroviral therapy and its correlates among HIV infected pediatric patients in Ethiopia

**DOI:** 10.1186/1471-2431-8-53

**Published:** 2008-12-06

**Authors:** Sibhatu Biadgilign, Amare Deribew, Alemayehu Amberbir, Kebede Deribe

**Affiliations:** 1 Jimma University, Public Health Faculty, Department of Epidemiology and Biostatistics, Jimma, Ethiopia; 2 Addis Ababa University, School of Public Health, Butajira Birth cohort project, Addis Ababa, Ethiopia; 3 Fayyaa Integrated Development Association-NCMI, PEPFAR-New Partners Initiative, P.O. Box 19715 Addis Ababa, Ethiopia

## Abstract

**Background:**

The introduction of combination antiretroviral therapy (ART) has resulted in striking reductions in HIV-related mortality. Despite increased availability of ART, children remain a neglected population. This may be due to concerns that failure to adhere appears to be related to continued viral replication, treatment failure and the emergence of drug-resistant strains of HIV. This study determines the rates and factors associated with adherence to Antiretroviral (ARV) Drug therapy in HIV-infected children who were receiving Highly Active Antiretroviral Therapy (HAART) in Addis Ababa, Ethiopia in 2008.

**Methods:**

A cross-sectional study was conducted in five hospitals in Addis Ababa from February 18 – April 28, 2008. The study population entailed parents/caretaker and index children who were following ART in the health facilities. A structured questionnaire was used for data collection.

**Results:**

A total of 390 children respondents were included in the study with a response rate of 91%. The majority, equaling 205 (52.6%) of the children, were greater than 9 years of age. Fifty five percent of the children were girls. A total of 339 children (86.9%) as reported by caregivers were adherent to antiretroviral drugs for the past 7 days before the interview. Numerous variables were found to be significantly associated with adherence: children whose parents did not pay a fee for treatment [OR = 0.39 (95%CI: 0.16, 0.92)], children who had ever received any nutritional support from the clinic [OR = 0.34 (95%CI: 0.14, 0.79)] were less likely to adhere. Whereas children who took co-trimoxazole medication/syrup besides ARVs [OR = 3.65 (95%CI: 1.24, 10.74)], children who did not know their sero-status [OR = 2.53 (95%CI: 1.24, 5.19)] and children who were not aware of their caregiver's health problem [OR = 2.45 (95%CI: 1.25, 4.81)] were more likely to adhere than their counterparts.

**Conclusion:**

Adherence to HAART in children in Addis Ababa was higher than other similar setups. However, there are still significant numbers of children who are non-adherent to HAART.

## Background

HIV/AIDS is one of the most destructive epidemics the world has ever witnessed. In 2007 an estimated 33.2 million people were living with HIV (PLHIV) worldwide, while 2.5 million of these people were children under 15 years old. Furthermore, 420,000 Children under 15 years were newly infected with HIV in 2007. Nearly 90% of all HIV-positive children live in sub-Saharan Africa [1]. About 780,000 were estimated to be in need of antiretroviral therapy [2]. The most efficient and cost effective way to tackle pediatric HIV globally is to reduce Mother to Children Transmission (MTCT). However, every day there are nearly 1200 new infections in children under 15 years of age with more than 90% of these occurring in the developing world and most being associated with MTCT [3].

Trials in the United States and Western Europe have demonstrated that HAART is effective in suppressing HIV viral replication and reversing immunodeficiency in children [4,5]. The result has been a reduction in pediatric hospital admissions and a decrease in morbidity and mortality due to HIV/AIDS [6-9]. The introduction of combination ART including protease inhibitors has resulted in striking reductions in HIV-related mortality [10-12]. However, these therapeutic regimens are very complex, often requiring that patients take numerous pills multiple times a day with specific timing and food restrictions or requirements. Failure to adhere very closely to the regimens appears to be related to continued viral replication, treatment failure and the emergence of drug-resistant strains of HIV [13-15].

In sub-Saharan Africa, AIDS has become one of the leading causes of mortality among children under the age of five years. Despite increased availability of ART, children remain a neglected population group [16]. However, many factors can affect the ability of HAART to suppress viral replication, including low potency of one of the drugs in the combination, viral resistance, inadequate drug exposure and inadequate adherence to therapy. The major factor determining the Success of HAART is sustainable and optimum adherence to therapy [17] as poor adherence increases the risk of virologic failure and viral resistance [18]. Therefore this study determines the rates and factors associated with adherence to ARV therapy among HIV-infected children who are receiving HAART in Addis Ababa, Ethiopia.

## Methods

### Study area

The study was conducted in selected hospitals in Addis Ababa city, the Capital City of the Federal Democratic Republic of Ethiopia. As of August 2007 the total number of children on ART in Ethiopia was 3865 [19]. The study was carried out in selected ART units in Addis Ababa Hospitals including Black Lion, St. Peter, Yekatit 12, Zewditu, and ALERT Hospitals. Among these, Black Lion Hospital is a teaching hospital under the Federal Ministry of Education. St. Paul Hospital is a General Specialized Hospital under the Federal Ministry of Health. St. Peter Hospital is a Generalized Hospital for TB treatment and they recently opened an ART clinic for adult and children. ALERT Hospital is also under the Federal Ministry of Health. Yekatit 12 and Zewditu Hospitals are under the Addis Ababa Regional Health Bureau, known to serve most of the patients on ART follow-up. This study was conducted from February 18 – April 28, 2008. The study design was a facility-based cross-sectional study.

### Participants

The source populations were children who had been taking ARV and were on follow up in the ART units of the selected hospitals during the study period. The study population included sampled children who had been taking combination ARV medications who were on follow-up as well present during the data collection period. Children who fulfilled the following criteria were included in the study: Receiving continuous antiretroviral therapy for the last 12 weeks before study in the selected hospital and the caregiver (parents or guardians) was counseled on the importance of drug adherence and on how to recognize common adverse drug reactions associated with antiretroviral drugs. The exclusion criteria were: children who were terminally ill, age less than 3 months or greater than 14 years and a caregiver in a moribund state. The sample size was calculated using two proportions sample size formula using Epi Info (CDC, Atlanta, U.S.A., 2005) 6.04 statistical package. The following parameters were used to calculate the sample size: proportion of adherence (P1) among male children 33%, proportion of adherence (P2) among female children 20% [20], 95% CI and 80% power. This gave a sample size of 393. Adding 10% for non-response rate, the total sample size was 433. A simple random sampling technique was employed to identify the study units using the ART unique numbers from the registration book in each hospital.

### Measurements

Data were collected by structured pre-tested questionnaire which was adopted from different studies [9,16-18]. The questionnaire was originally developed in English translated to Amharic and back translated to English by a translator who was blind to the original questionnaire. The content of the questionnaire included: socio-demographic characteristics (age of the child, sex of the child, age of the caregiver, ethnicity, educational status, occupational status of the caregiver); socio-economics conditions (monthly income, financial support for the child, grant for the child); medication related factors; health care delivery systems related factors including access to care, disclosure status of the children; perception related to the services, diagnosis, referral and treatment, and medication administration. Attitude was measured using a previously determined scale on attitude [16] where a set of 12 Likert scale questions addressed the attitude of the caregiver towards adherence. The independent variables were socio-economic status, socio-demographic factor, clinical characteristics (clinical stage of the children (World Health Organization stage I-IV) and CD4 cells/mm3 count of the children), perception related to the services (diagnosis, referral and treatment), medication administration including dosing. The dependent variable was caregivers' report adherence to ART in the past 7 days. A total of three days of intensive training was given to all supervisors and data collectors. The interview was conducted in private room to create an atmosphere of empathy and confidence. Medical charts were reviewed to collect clinical and immunological markers. The principal investigator and three general practitioners supervised the data collection process.

### Data analysis and processing

Data entry and analyses were carried out using SPSS version 12.0.1 statistical packages. First, descriptive statistics were carried out to explore the socio-demographic characteristics of the respondents, the adherence rate and clinical characteristics of the children. To find association between the exposure variables and adherence, bivariate analysis was done. To control the effect of confounding variables, stepwise logistics regression was done. Variables which showed statistical significant association (P < 0.05) in the bivariate analysis were included in the final model. To ensure quality of the data, pre-testing of the questionnaire was undertaken in 5 percent of the sample size in similar setups before the actual data collection took place. The final version of the questionnaire was used for the data collection. The overall activity was monitored by the principal investigator. There were strict supervisions during data collection. One trained data clerk entered and cleaned the data.

### Operational Definitions

Adherence – a child is said to be adherent if he/she missed no more than one dose (took more than 95% of the prescribed doses correctly) for one week prior to the study.

Primary caregiver – a person who has consistently assumed responsibility for the housing, health, or safety of the child (individuals who administered the child medication daily and bringing the child for clinic appointments).

Attitude – twelve Likert Scale attitude questions were presented to respondents. One variable was computed using the sum of the 12 questions. If respondents score more than 36, they were labeled as having favorable attitude. Unfavorable attitude was considered as a score less than 36.

### Ethical consideration

Ethical clearance was obtained from Institutional Ethical Review Committee of Jimma University and Ethical clearance Committee of Addis Ababa Health Bureau. An official letter of co-operation from the above organization and Federal Ministry of Health (FMOH) was given to respective hospitals. Caregivers gave verbal informed consent for participating in the study.

## Results

### Socio-demographic and economic characteristics

A total of 390 children respondents were included in the study with response rate of 91%. The majority 205 (52.6%) of the children was above 9 years. The median age of the children was 9 years (range: 1 to 14 years). Fifty five percent of the children were girls. Most of the study participants were Amhara (53%) and Oromo (24%) by ethnicity. A majority (76.2%) of the caregivers were orthodox by religion. Concerning the educational status of the caregiver, 176, or 45.1%, were in primary school. Regarding occupation, 103 (26.4%) were daily laborers and 80 (20.5%) were working as a government employee. One hundred seventy four (44.6%) of the caregivers were married and 138 (35.4%) were widowed. Three hundred seventy (94.9%) of the respondents were the primary caregivers of the child. Two hundred forty (61.5%) of the primary caregivers were biological parents of the children. The remaining were non-biological parents. The study subjects were on HAART for a mean and median duration of 20 and 24 months (12 to 48 month). One hundred sixty-two (67.5%) of the biological parents serving as a caregiver were the biological mother of child (See table 1).

**Table 1:** Demographic and social characteristics of caregiver and children in Addis Ababa, Ethiopia [N = 390], April 2008

Variable	Frequency(percentage)		p-value
	
	Adherent	Non-adherent	
Age (years)			0.672
<3	14(4.1)	1(2)	
3–5	39(11.5)	4(7.8)	
6–8	111(32.7)	16(31.4)	
> = 9	175(51.6)	30(58.8)	
Sex of the child			0.347
Boy	149(44)	26(51)	
Girl	190(56)	25(49)	
Religion of the caregiver			0.607
Orthodox	261(77)	36(70.6)	
Catholic	18(5.3)	3(5.9)	
Protestant	37(10.9)	8(15.7)	
Muslim	16(4.7)	2(3.9)	
Others *	7 (2.1)	2(3.9)	
Occupational status of the caregiver			0.343
Farmer	0(0)	4(7.8)	
Merchant	50(14.7)	5(9.8)	
Governmental employee	52(15.3)	6(11.8)	
NGO employee	28(8.3)	1(2)	
Day laborer	84(24.8)	19(37.3)	
Jobless/house made	71(20.9)	9(17.6)	
Others^*£*^	54(15.9)	7(13.7)	
Marital status of the caregiver			0.198
Single	36(10.6)	2(3.9)	
Married	154(45.4)	20(39.2)	
Divorced	32(9.4)	8(15.7)	
Widowed	117(34.5)	21(41.2)	
Educational status of the caregiver			0.107
Unable to read and write	75(22.1)	19(37.3)	
Primary (1–8)	157(46.3)	19(37.3)	
Secondary (9–12)	62(18.3)	6(11.8)	
Diploma and above	45(13.3)	7(13.7)	
Are you the primary caregiver			0.083
Yes	320(94.4)	51(100)	
No	19(5.6)	0(0)	
Who is the primary caregiver responsible for the child?			0.296
Biological parents	212(62.5)	28(54.9)	
Non-biological parents	127(37.5)	23(45.1)	
Child HIV status disclosure			0.001
Yes	51(15)	17(33.3)	
No	288(85)	34(66.7)	
Health care Provider's estimate of adherence			0.001
Good	295(87)	32(62.7)	
Fair	9(11.5)	11(21.6)	
Poor	5(1.5)	8(15.7)	
Monthly income in ETB			0.154
<105	235(69.7)	42(82.4)	
106–150	45(13.4)	5(9.8)	
151–262	57(16.9)	4(7.8)	
Do you know when the child start treatment			0.559
Yes	296(87.3)	46(90.2)	
No	43(12.7)	5(9.8)	
Duration of treatment in months			0.278
≤ 12	100(29.5)	21(41.2)	
13–24	185(54.6)	21(41.2)	
24–36	44(13)	8(15.7)	
≥ 36	10(2.9)	1(2)	

Care givers Attitude about Child ART			0.770

Favorable	330(97.3)	50(98)	

Unfavorable	9(2.7)	1(2)	

Others *--

Local, Jehovah witness

Others^*£ *^– secretary, cashier, carpenter

Exchange rate 1 USD = 9.6 Ethiopian Birr (ETB).

A large proportion of caregivers, equaling 277 (71%), mentioned that no one had helped the child financially. Fathers (6.4%) and local NGOs (6.4%) were responsible to help some of the children. Of the 390 caregivers, 21.8 percent received a childcare grant. About 277 (71.4%) of the respondents had household income levels below 500 Eth. Birr per month (Exchange rate 1 USD = 9.6 Ethiopian Birr (ETB)). (Data not presented)

### Clinical Markers of the Participants

Most of the children (193 [49.5%]) were in stage III based on WHO classification. The mean and median initial CD4 count were 273.28 cells/mm3 and 203.00 cells/mm3 respectively and 576.92 cells/mm3 and 497.50 cells/mm3 were the latest CD4 counts. One hundred ninety-four (49.7%) of the children had a CD4 count of <200 cells/mm3 at the start of the treatment. A similar number of children had CD4 count of > = 500 cells/mm3. One hundred eleven children (30%) had been taking the Stavudine (d4T)/Lamivudine (3TC)/Nevirapine (NVP) based regimen. About 115 (27%) of the children were taking Zidovudine (AZT)/3TC/NVP and AZT/3TC/(Efavirenz) EFV for each regimen (See Table 2).

**Table 2:** Clinical markers of HIV infected children in Addis Ababa, Ethiopia, [N = 390] April 2008

Variable	Frequency(percentage)		p-value
	
	adherent	Non-adherent	
WHO Clinical stage of HIV disease			0.226
Stage I	14(4.13)	1(2)	
Stage II	102(30.1)	9(17.6)	
Stage III	163(48.07)	30(58.8)	
Stage IV	60(17.7)	11(21.6)	
CD4 counts at start of treatment			0.377
<200	164(48.4)	30(58.8)	
200–499	131(38.6)	16(31.4)	
> = 500	44(13)	5(9.8)	
Current CD4 count			0.073
<200	45(13.3)	12(23.5)	
200–499	119(35.1)	20(39.2)	
> = 500	175(51.6)	19(37.3)	
Regimen recommended			0.105
4a = d4T/3TC/NVP	103(30.4)	16(31.4)	
4b = d4T/3TC/EFV	53(15.6)	9(17.6)	
4c = AZT/3TC/NVP	97(28.6)	7(13.7)	
4d = AZT/3TC/EFV	86(25.4)	19(37.3)	

d4T = Stavudine, 3TC = Lamivudine, NVP = Nevirapine, AZT = Zidovudine, EFV = Efavirenz

### Knowledge, Perceptions and Attitude about ARV Medications Administration

Seventy percent of the participants (caregivers) had received information about ARV treatment from the hospitals (Government and Private). Overall, the participant attitudes on administration of ARV medication to the children were favorable in 380 (97.4%) of the respondents with the 12 points Liker scale instrument (See Table 1).

### Medication Administrations and Adherence

**Figure 1:**
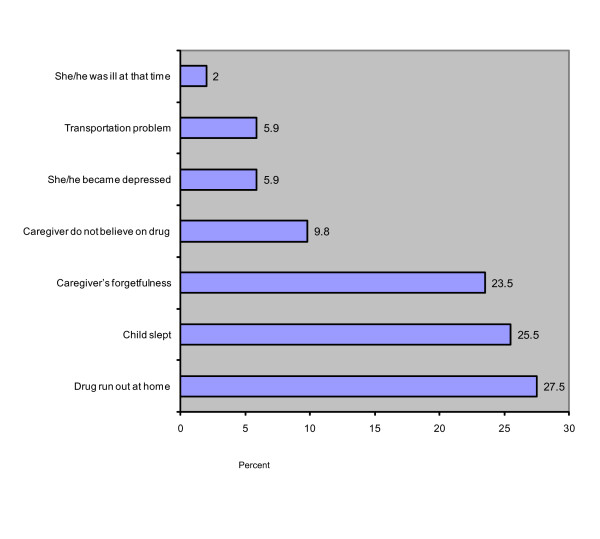
Reasons for missing dose in children who were on HAART in Addis Ababa, Ethiopia, April 2008.

Caregivers used different mechanisms as reminders to administer medications on time; the most common reminder was cellular phone alarm systems (25.4%) followed by watches/clocks (46.4%). A total of 51 children (13.1%) omitted at least one dose of antiretroviral drugs in the past 7 days before the survey (See Table 3). For those who missed a dose or more in the last 7 days, the common reasons were lack of medication (27.5%), child slept (25.5%) and forgetfulness to give the drugs (23.5%) (See Figure 1).

**Table 3:** Caregivers' report adherence rate of HAART among children in Addis Ababa, Ethiopia, April 2008

Days	Adherent No(%)	Non adherent No (%)	Total
Today	383(98.2%)	7(1.8%)	390(100%)
Yesterday	378(96.9%)	12(3.1%)	390(100%)
Past 3 days	363(93.1%)	27(6.9%)	390(100%)
Past 7 days	339(86.9%)	51(13.1%)	390(100%)

### Factors Associated With Pediatric Adherence to Antiretroviral Therapy

After controlling the effects of other variables, five variables were found to be significantly associated with adherence to ART in children: As shown in Table 4, children whose parents did not pay a fee for treatment were 61.3% less likely to adhere for ART than those who paid for their medication [OR = 0.39 (95%CI: 0.16, 0.92)]. Similarly, children who had ever received any nutritional support from the clinic [PEM scheme, etc.] were 66.3% less likely to adhere to ART than those who did not get the nutritional support [OR = 0.34 (95%CI: 0.14, 0.79)]. Children who took co-trimoxazole medication/syrup besides ARVs were 3.64 times more likely to adhere than those who did not take the co-trimoxazole [OR = 3.65 (95%CI: 1.24, 10.74)]. Children who did not know their sero-status were 2.53 times more likely to adhere for the treatment of ARV than children who did know their sero-status [OR = 2.53 (95%CI: 1.24, 5.19)]. Children who were not aware of their caregiver's health problem were 2.44 times more likely to adhere than children who had perceived awareness about the illness of the caregiver [OR = 2.45 (95%CI: 1.25, 4.81)].

**Table 4:** Independent predictors of adherence to ART among children in Addis Ababa, Ethiopia (n = 390), April 2008

Variable	Adherence Status		Crude OR (95%CI)	P-value	Adjusted OR (95%CI)	P-value
					
	Non-adherent	Adherent				
Medication charge for child treatment before ARV intake						
YES	7	104	1.00	0.016	1.00	
NO	44	235	0.36(0.16,0.83)		0.39(0.16,0.92)	0.033
Co-trimoxazole intake beside ARV						
Yes	44	324	3.44(1.33,8.93)	0.011	3.65(1.24,10.74)	
No	7	15	1.00		1.00	0.019
Perceived awareness of the child on health problem of caregiver						
Yes	20	63	1.00		1.00	0.009
No	31	276	2.826(1.51,5.28)	0.001	2.45(1.25,4.81)	
The child knows his/her sero-status						
Yes	17	51	1.00	0.002	1.00	0.011
No	34	288	2.824(1.47,5.43)		2.53(1.24,5.19)	
Received nutritional support from the clinic						
Yes	44	216	0.28(0.12,0.64)	0.003	0.34(0.14,0.79)	0.013
No	7	123	1.00		1.00	

OR = Odds Ratio, CI = Confidence Interval

## Discussion

This paper tries to examine the different variables associated with child adherence to antiretroviral therapy for in Ethiopia. Clinical record review, immunological markers and psychological and medication-related factors were assessed along with the caregiver characteristics to determine the predictors of adherence.

Adherence is a special issue in pediatrics not only because of social situations but also because many of the drugs are not child friendly [21]. This study found an estimated prevalence of caregivers' report of adherence to antiretroviral treatment to be 93.1% in 3 days and 86.9% in a 7-day recall period. Adherence rate in other studies ranged from 26% to 97% [22-30]. The possible explanations for the greater adherence in our study might be that the majority of the children started ART recently, the children were taking medication with a twice-daily dosing schedule, or the children and caregivers were given strict adherence counseling sessions before starting ART in the hospitals and the majority of caregivers (97.4%) had a favorable attitude toward administration of ARV to children.

In this study children whose caregivers paid for medication before ART were more adherer than those who did not pay. Hence, people tend to give value for their health when they spend money for treatment of their illness. Similarly, studies [31,32] revealed that costs have an implication for adherence. However, it varies at different stages of HIV infection, such that patient's who present late, especially if more immuno-suppressed, use more services at greater cost than those who are less immuno-suppressed. An increasing adherence rate was observed in children with more advanced HIV [33]. Again, a similar pattern has been reported in adults [34,35].

One of the entry points that complicated the issue of adherence for HIV infected children is the issue of disclosure of HIV status to the child [36]. Studies showed that complete parental disclosure helps to motivate HIV-infected children to adhere to their daily medical regimen. It enables children to understand HIV infection and to make sense of disease-related experiences and the importance of adherence [37,38]. In this study, however non-disclosure had a significant relevance for adherence to the recommended regimen. It is consistent with other studies, which showed no effect of disclosure on adherence to ART [22,39], which is also the case in the Multicenter National Study in Italy [33].

Interestingly, those who took co-trimoxazole prophylaxis besides taking ARV medications were more likely to adhere than those who did not use the treatment. Prophylaxis is probably at least as important as ART in preventing the onset of AIDS in children [40,41]. It is also supported by a study which revealed daily co-trimoxazole lays the groundwork for medication adherence by the patient and the establishment of reliable drug distribution systems [42]. Nutrition interventions can help to optimize the benefits of ARVs and may increase compliance with treatment regimens, both of which are essential to prolonging the Lives of People Living with HIV (PLHIV) and preventing the MTCT [43]. However; in our study receiving nutritional support from the clinic had an inverse relationship with adherence to ART therapy. In contrast to our finding, it is reported that one of the significant barriers to adherence was insufficient food and patients reported that lack of food prevented or delayed them taking their medication [44]. Provision of food and micronutrients has been shown to improve outcomes [45-47]. In a South African study, many families spend more than 50% of their household income on food and food production and wage earnings were adversely affected when an adult has AIDS [48]. In the same line, poverty factors such as food insecurity and user fees for medical care, posed more significant barriers to adhering to long-term therapy than a patient's individual behavior. In a pilot program in Zambia on nutritional supplementation for food insecure patients on ART, it was shown that patients receiving food were on average 2.4 days late for their pharmacy appointments, whereas patients not receiving food were on average 3.4 days late each month. Patients receiving food were significantly adhered to pharmacy visits than patients not receiving food [49]. Surprisingly, in our study, adherence rates were significantly lower in those who received nutritional support than in those who did not. The inverse relationship between adherence to HAART and nutritional support is somewhat worrisome and needs to be investigated further in order to plan interventions in HIV infected children.

This study had some limitations and strengths. The main limitation of this study was recall bias. There is no gold standard assessment of adherence. In this study, adherence was measured using self-reports from the caregivers, which tends to overestimate the prevalence of adherence. The cross-sectional nature may hinder the ability to exactly identify the predictor of adherence, unlike a longitudinal design. Caregivers might be prone **to **social desirability bias responding inappropriately to the counselors. Adherence classification cutoff points may not be perfect in different setups to compare and contrast the finding. Despite the above limitations, the study had several strengths, including using a relatively large sample size, inclusion of several sites, use of more than one method of adherence assessment and inclusion of several variables.

## Conclusion

From this research finding, it is concluded that caregivers' report adherence to HAART in children in Addis Ababa was 93% in the past 3 days and 87% in the past 7 days before the date of interview and it is acceptably higher than other similar setups. Children whose parents did not pay a fee for treatment, children who had ever received any nutritional support from the clinic were less likely to adhere. While children who took co-trimoxazole besides ARVs, children who did not know their sero-status and children who were not aware of their caregiver's health problem were more likely to adhere than their counterparts. To encourage child adherence to ART, using co-trimoxazole prophylaxis along with the delivery of ART might help. Further research is recommended to explore the relationship between disclosure and adherence and the effect of nutritional support on adherence.

## Competing interests

The authors declare that they have no competing interests.

## Authors' contributions

SB conceived and designed study, performed analysis and interpretation of data and drafted the manuscript. AD assisted with the design, interpretation of data and the critical review of the manuscript. AA assisted with the conception and designing the study and critically reviewed the manuscript. KD conceived and designed the study, critically reviewed the manuscript. All authors read and approved the final manuscript.

## Pre-publication history

The pre-publication history for this paper can be accessed here:


